# The problem of moral obligation to preserve or erase memories in trauma treatment

**DOI:** 10.1186/s13010-025-00203-0

**Published:** 2025-11-06

**Authors:** Junjie Yang

**Affiliations:** 1 Department of Philosophy, Peking University, Beijing, China; 2 Department of Philosophy, Yale University, New Haven, USA

**Keywords:** Trauma, Memory modification, PTSD, Transformative experience, Neuroethics

## Abstract

People who have experienced traumatic events often suffer from the burden of painful memories. Recent advances in neuropharmaceuticals and neurotechnologies have enabled the modification and even erasure of traumatic memories, raising both therapeutic hopes and ethical concerns. One view argues that individuals have a moral obligation to preserve traumatic memories; therefore, erasing such memories amounts to an evasion of moral obligations and is therefore unacceptable. However, neither deontological ethics nor rule consequentialism can justify the claim that patients have an obligation to preserve their traumatic memories. In fact, memory erasure, as a transformative experience, situates individuals within a context of decision-making under uncertainty, thereby highlighting their moral obligations to themselves. Trauma survivors may seek memory erasure technologies as a way of honoring their moral obligations to their past, present, and future selves. In this sense, such interventions may be regarded as morally permissible.

## Introduction

Trauma exposure has become a global mental health issue, imposing a substantial and lasting burden on individuals’ lifelong physical and psychological well-being, as well as on public health systems [1–3]. In the treatment for post-traumatic disorder (PTSD), beyond traditional psychological interventions such as cognitive behavioral therapy (CBT), neurological and neurosurgical approaches aimed to blunt or even erase traumatic memories have recently demonstrated significant clinical potential. Currently, neuropharmaceuticals such as beta-adrenergic blockers can be used to attenuate the emotional valence of distressing memories, while neurostimulation technologies, such as deep brain stimulation (DBS), have shown the capacity to suppress traumatic memories [4–6]. Having been extensively tested in preclinical models and cautiously introduced into clinical practice, these memory interventions exhibit emerging therapeutic promise for trauma. Some emerging approaches, such as optogenetics, which use light to directly manipulate neurons and inhibit memory formation or retrieval, have also demonstrated promising potential for clinical translation [7]. These methods can all be categorized as memory erasure technologies (METs), expanding the therapeutic possibilities for PTSD patients who suffer from traumatic memories [8].^1^

Some ethicists have questioned METs for their uncertain safety, risk of irreversible harm, and implications for personal identity [9–11]. One prominent objection, based on the considerations of moral duty and obligation, challenges the permissibility of therapeutic memory erasure. This argument, referred to as the Obligation Evasion Argument, asserts that all people have compulsory moral obligations to remember and not to forget traumatic memories, and therefore, treating trauma through METs constitutes an evasion of moral obligation and is thus morally unacceptable.

This view appears plausible, as we often speak of a “duty to remember” in everyday moral thinking. However, it is important to consider how the terms *obligation* and *duty* are employed in ethical analyses of METs. We commonly say that “it is obligatory for *A* to do *X*” and “it is *A*’s duty to do *X*” [12]. These two terms are often used interchangeably, although some philosophers argue for a stricter distinction, arguing that obligation involves voluntariness and consent, whereas duty does not. Rawls points out that natural duties are those that everyone must bear unconditionally, such as the duty to avoid causing harm [13]. As Hart argues that “obligations are voluntarily incurred, whereas duties arise from position, status, or role.” [14] Additionally, some institutional rules, such as research integrity or procedural justice, are sometimes regarded as obligations or duties that must be upheld, stemming from the demands of public reason. This article treats duty as unconditional and obligation as conditional, and seeks to address the following questions: What does it mean to have a moral obligation to remember and not to forget? What is the source of our moral obligation to preserve traumatic memories? And does erasing such memories constitute an evasion of moral obligation?

Before engaging in the ethical discussion of MET, it is necessary to clarify the moral significance of remembering and forgetting. Memory is a domain in which the personal and the social are deeply intertwined, where individual narratives are continually shaped through relational contexts [15]. On the one hand, the mental phenomenon of memory can be regarded as residing within one’s brain, preserving a certain opacity to others [16]. On the other hand, memory also has a public nature, extending beyond individual boundaries in social interactions and serving as an important link for maintaining social relationships [17]. For example, we rely on memory to fulfill commitments to others and respond to their expectations of us. As such, memory “holds value not just for the ‘owner’ of the memory but for other individuals or society as a whole as well” [18]. Consequently, remembering is endowed with moral significance. Although remembering, like forgetting, can at times be involuntary, it is, intuitively, not something over which one may exercise moral discretion [19]. Forgetting something that one has a duty to remember warrants moral criticism. For instance, we seem to have an obligation to remember those who have cared for us, and we should remember the agreement that is important for both. However, whether there exists a general obligation to remember traumatic events remains contentious. Some ethicists argue that using MET to eliminate traumatic memories constitutes an act of evading moral obligation, and therefore oppose the use of such technologies in trauma treatment. These objections may be classified according to two different theoretical approaches to moral obligation: *deontological ethics* and *rule consequentialism* [7, 18–22]:

1. **Deontological ethics**: We inherently bear certain *compulsory* duties to do what is morally right, which includes fulfilling the moral obligation to preserve traumatic memories. Erasing traumatic memories constitutes an evasion of this obligation, and therefore, we should not erase traumatic memories.
2. **Rule consequentialism**: Traumatic memories hold *social value*, and the principle of maximizing social value mandates the moral obligation to preserve traumatic memories. Erasing traumatic memories is an evasion of this obligation to maximize social value, and therefore, we should not erase traumatic memories.

However, in this article, I argue that neither position adequately establishes a moral obligation to preserve traumatic memories. Instead, I argue that the issue of whether we have a moral obligation to preserve or erase traumatic memories should be examined from the perspective of the obligations to oneself. While individuals may find it difficult to fully anticipate, both epistemically and personally, the consequences of memory erasure, trauma survivors burdened by painful memories ought to recognize their self-directed moral obligations. To be more specific, this article rejects the idea of a universal duty to preserve traumatic memories and instead defends a context-dependent obligation tied to the individual’s moral role in transformative experiences of using METs.

The structure of this paper is as follows. Section The deontological perspective on the obligation to preserve traumatic memories challenges deontological justifications for a duty of knowledge, assistance, and justice to preserve traumatic memories; Sect. The rule consequentialist perspective on the obligation to preserve traumatic memories rejects rule-consequentialist defenses based on public safety, psychological resilience, or collective memory; and Sect. Obligations to oneself from the perspective of transformative experience, drawing on the notion of transformative experience, develops a self-directed account, arguing that METs can be morally permissible as they enable trauma survivors to fulfill obligations to their future, present, and past selves. Examining whether individuals facing the choice of undergoing MET are morally obliged to preserve traumatic memories addresses fundamental philosophical questions concerning autonomy, consent, and personal identity, while also providing valuable ethical insights into the current application of neurotechnological interventions for psychiatric disorders.

## The deontological perspective on the obligation to preserve traumatic memories

Within deontological ethics, we seem to bear an inescapable moral obligation to remember on behalf of others. We ought to remember and must not forget because doing so is the right thing per se, and this duty carries an obligatory force. As the President’s Council on Bioethics stated:

Surely, we cannot and should not force those who live through great trauma to endure its painful memory for the benefit of the rest of us. But as a community, there are certain events that we have an obligation to remember—an obligation that falls disproportionately, one might even say unfairly, on those who experience such events most directly [23].

This deontological stance may entail a range of distinct moral requirements. For instance, the obligation to remember might involve making every effort to preserve one’s memories, merely refraining from active forgetting, or maintaining only the semantic content of memories while disregarding their associated emotional responses. In other words, memory erasure and preservation are not strictly binary. Yet the deliberate use of memory erasure technologies presents a more clearly defined target for deontological critique. Although this ethical stance requires individuals who have endured severe trauma to confront painful recollections and may, in some cases, appear harsh, they nonetheless bear a moral obligation to refrain from employing METs.

Previous discussions have grounded this deontological justification in the social roles that individuals universally occupy within society [24]. As bearers of memory, we may act as knowers, helpers, and upholders of justice, and promoting knowledge, care, and justice are moral duties we ought to fulfill in society. Erasing traumatic memories would thus entail neglecting the duties of knowledge, assistance, and justice, rendering such intervention morally impermissible [7, 11, 20–22, 25]. However, as this section will demonstrate, this perspective faces difficulties.

### The duty of knowledge

One justification for the moral obligation to preserve traumatic memories lies in the epistemic value of memory, as memories provide evidence that can constitute or improve knowledge. We have a duty to preserve memories that contribute to understanding, “not just for oneself but often also for others” [11]. In the context of trauma treatment, memory erasure eliminates this evidentiary value: by selectively removing memories, individuals not only lose access to their own evidence but also undermine the collective knowledge others depend on. It is an outcome that deontological ethics cannot permit.

Imagine a key eyewitness who was hiding in a closet at the crime scene, unnoticed by the perpetrator, making them the only person who witnessed the crime. This horrible experience caused the eyewitness trauma, and she wishes to erase the memory through treatment. However, the perpetrator is still at large and needs to be identified, and the eyewitness’s memory is crucial evidence [7, 22, 26]. In this case, from the perspective of the duty of knowledge, the eyewitness has an obligation to preserve this traumatic memory as evidence to help the police apprehend the criminal. Therefore, choosing to erase a traumatic memory solely to avoid recalling painful experiences, and thus to evade the moral obligation to remember, cannot be morally justified.

From the standpoint of the duty of knowledge, the epistemic obligations to preserve traumatic memories rest on the assumption that memory serves as a form of evidence. However, this premise is problematic. First, epistemic duties require us to provide “reliable” evidence, but memory is not always “reliable.” Many psychological studies on false memories indicate that eyewitness memories are often susceptible to bias, emotion, and misleading information, which can lead to their reconstruction or alteration [27, 28]. This unreliability weakens the foundation for memory as evidence [29]. Some may argue that, in courtroom settings, a witness’s unreliable memory can be offset by other mechanisms—such as a jury’s evaluation or corroborating evidence—thereby preserving the basic evidential function of testimony. However, not all events can be supported by reliable corroborating evidence, and even when such evidence exists, its interpretation still depends on memory as a form of second-order evidence, thereby risking an infinite regress concerning the reliability of the supporting evidence itself. In other words, although it must be acknowledged that absolutely reliable evidence can never be achieved through memory, this very fact also implies that the epistemic obligation cannot be fulfilled merely by preserving memory, nor does the fulfillment of that obligation necessarily depend on memory itself.

Secondly, even if memories possess epistemic value, this does not necessarily entail that individuals are required to preserve their own firsthand memories personally. The epistemic duties arising from the evidential function of memory may, in principle, be satisfied through externalized means, such as recorded testimony, sworn statements, or certified composites. For instance, after a witness creates a composite sketch of the suspect or records an oral testimony with the assistance of the police, the evidence can be effectively transferred from the individual’s mental memory to the physical world, entrusted to other key figures in the legal system [30]. Additionally, electronic technologies can also help us store memories, contributing to our “extended memory” [31]. There have already been attempts to obtain testimony from witnesses remotely with the secure encryption, allowing lawyers to preserve evidence from parties as soon as possible after an event, which can serve as admissible evidence in court [32]. This preserves the evidentiary function of memory, meaning that individuals may intervene in their traumatic memories without necessarily personally fulfilling the obligation to remember evidence.

Moreover, the evidentiary attribute of memory seems to suggest that the semantic aspects of memory are of greater concern for moral obligations. From this viewpoint, erasing the emotional memory associated with an event may not necessarily diminish one’s duties of knowledge. To be specific, episodic memory contains both semantic content and affective tags, and when memory is employed as evidence, the removal of certain emotional elements, as in the mechanism by which drugs such as propranolol may operate, may not compromise its basic function in factual testimony. For example, when an eyewitness identifies a suspect, “I remember he was about six feet tall” can serve as evidence, but “I remember feeling very scared when I saw him” may not be considered evidence. If the valuable information lies only in the semantic knowledge of the event, then erasing the emotional content of a memory while preserving its semantic components might suffice to fulfill the duty of knowledge [33]. Consequently, using neuropharmacological drugs such as propranolol to blunt certain emotional memories may not necessarily threaten the memory obligation.

However, some may argue that the emotional dimension of memory cannot be overlooked, as it profoundly shapes individuals’ understanding and attitudes toward events and possesses significant epistemic value [34, 35]. For instance, memories stripped of their affective valence may weaken a witness’s motivation to testify or to uphold justice. However, a symmetrical consideration is also necessary: when the emotional burden of memory becomes overwhelming and leads to severe psychological distress, preserving such memories may, in fact, undermine their evidential function and thereby hinder individuals from fulfilling their corresponding duty of knowledge. In extreme circumstances, such as witnessing mass violence, holocaust, or experiencing severe abuse, individuals may endure intense psychological distress. In these situations, requiring individuals to preserve emotional memories as evidence may not only fail to achieve the fulfillment of the memory obligation but could also constitute an excessive moral demand [20].

### The duty of assistance

The second reason for the moral obligation to preserve traumatic memories is that memory fosters empathy and care in society, and we should fulfill this mutual duty.^2^ Through memory, we are able to understand others, cultivate empathy, and promote the fulfillment of moral duties. As Margalit pointed out, the prerequisite for our memory obligations is our desire to establish caring relationships with others [24].

Erasing traumatic memories may be an evasion of this duty of assistance. Wasserman argues that painful memories are crucial in shaping our conscience, as they help us remember past mistakes, harm, and lessons, thereby stimulating greater moral sensitivity [37]. In other words, suppressing or erasing painful memories may dull our sensitivity to both our own and others’ misfortunes, erode our capacity for empathy, and even lead to indifference toward suffering and cruelty [33]. Furthermore, some scholars argue that memory makes empathy possible, and by losing painful memories, we would no longer be able to truly perceive the suffering of others, making it difficult to engage in morally meaningful actions [38]. Liao and Wasserman note that forgiveness, as an important moral action, requires us to overcome anger or resentment based on our values while preserving memories of past experiences [39]. Erasing memories removes the possibility of such anger or resentment and, on a deeper level, undermines the ethical foundations of forgiveness, care, and reconciliation. Therefore, from the perspective of fulfilling the duty of assistance, we should preserve traumatic memories rather than erase them.

However, this deontological approach faces two main challenges. First, we may not need vivid and precise episodic memories to care for and assist others. Although some shared or comparable experiences may seem to facilitate empathy, this does not entail that such experiences must remain entirely untouched for empathy to be possible. Our mental states are marked by a degree of intersubjective opacity, yet we are still able to compassionately understand and respond to the situations of others. Nel Noddings emphasizes that care is fundamentally relational: what matters is the motivation to enter another’s frame of reference [40]. Similarity of memory alone does not provide such motivation. Moreover, if METs are to erase the emotional charge of a troubling experience while leaving intact the semantic knowledge that going through such distress was unpleasant, this could still enable individuals to fulfill the obligation of care. As Brendan Gaesser argues, semantic knowledge “could be used to guide or inform episodic simulations of helping events without relying on perspective taking.” [41] Thus, memory per se is not a necessary condition for care.

Second, pain does not necessarily serve as the basis for empathy or mutual assistance. While it might be argued that the loss of one’s ability to recall painful experiences in similar situations could indeed diminish empathetic responses to others’ suffering, the duty to assist is not inherently contingent upon such pain. In fact, such memories may, in some circumstances, obstruct the fulfillment of mutual assistance. For example, PTSD can lead to emotional numbing, detachment, and impaired interpersonal functioning, thereby making it difficult for individuals to carry out their obligations of care and support [42, 43]. By alleviating trauma-induced pain through the erasure of traumatic memories, individuals may recover from states of emotional numbness and motivational deficits, and, in turn, become better able to care for and assist others.

### The duty of justice

The duty of justice underscores that the preservation of traumatic memories is essential to the pursuit of social justice. For example, Paul Ricoeur argues that the duty of memory is inherently linked to justice: our memory duty lies in ensuring that justice is realized, by fulfilling this duty through feedback and transmission of all that we have received [44]. In addition, a view grounded in *corrective justice* holds that the duty to remember is an obligation owed to victims and survivors, which is a categorical imperative not to allow injustice to be forgotten [19]. If an individual chooses to erase traumatic memories, their understanding of the social significance of such memories may be altered, and their moral choices and value judgments related to the traumatic event could change. For example, the social bonds and relationships implicated in the traumatic event may become blurred or weakened, and the motivation to learn lessons from the experience, to remind society of injustice, or to promote social reform may be diminished. In particular, for victims of crimes such as sexual violence, erasing traumatic memories may attenuate their emotional responses. For instance, they may no longer “feel as bad after what happened to [them].” [45] This could, in turn, blur their attribution of responsibility to the perpetrator and diminish their inclination to seek justice. Overall, those who erase traumatic memories may no longer resist injustice in society, and such avoidance of the duty of justice is morally unacceptable [7, 46].

This duty of justice to preserve traumatic memories appears to be present in many contexts. In judicial situations, even though some offenders may be tormented by the horrific memories of their crimes and immersed in remorse, we seem to believe that these people should preserve such memories rather than evade the condemnation of their conscience through memory erasure. This is seen as necessary for upholding social justice. Particularly when offenders forget their actions, the absence of memory cues for remorse may lead them to commit crimes again [20]. For example, after committing her crime, Lady Macbeth endures endless psychological torment, repeatedly trying to “wash” the blood from her hands, believing that “all the perfumes of Arabia will not sweeten this little hand” [47]. She would rather drink a potion to erase her memories, but this would weaken her reflection on the crime she committed. Therefore, attempting to heal Lady Macbeth’s traumatic memories is misguided, as this pain itself is a necessary response to justice [9, 37]. In large-scale social events, the social duty of justice seems to demand that we should not erase the traumatic memories of significant events. For instance, although the memories of Holocaust survivors are filled with pain, they are seen as having a duty to preserve these memories in order to uphold justice and prevent society from repeating the mistakes of history [23].

However, the invocation of justice does not conclusively determine the moral impermissibility of erasing traumatic memories. First, forgetting traumatic memories does not inevitably represent an evasion of duty of justice. For example, in criminal jurisprudence, *restorative justice* emphasizes repairing the harm caused to victims, offenders, and the community. Remorse and regret grounded in traumatic memories may serve as a pathway to restoration, reflecting an offender’s moral reflection and signaling a lower risk of recidivism [48]. However, the mere retention of traumatic memories does not necessarily foster genuinely moral response to wrongful conduct. Some offenders may, through traumatic memories, come to recognize and regret their wrongful treatment of others. Yet the justification of punishment is weakened if such remorse stems not from an acknowledgment of past injustice, but merely from the perception that the punishment imposed is disproportionately harsh. Taking Lady Macbeth as an example again, although she is immersed in regret due to painful memories, her psychological breakdown does not transform into any form of compensatory justice action. Moreover, some might argue that the psychological suffering endured by offenders constitutes a form of *retributive justice*, in the sense that punishment is proportionate to the gravity of the offense, and that interfering with the memory structures sustaining such suffering would therefore be unjustified. Yet the justificatory foundation of retributive justice cannot rest solely on the pain of remorse as a sufficient reason for punishment, particularly when such suffering could yield more harm than good. When traumatic memories become rigidly fixed in offenders, they may even increase the likelihood of future offending [20]. In such cases, the preservation of traumatic memories paralyzes the individual in action, rather than enabling her to discharge the duties of justice more effectively.

Second, when individual wellbeing and social justice duties come into strong conflict, preserving traumatic memories may also fail to achieve the goal of justice. Opponents of memory erasure argue that victims of traumatic events must endure the pain of memory in order to fulfill collective duties. However, as Kolber asks, “[…] can the community, or even humanity as a whole, truly achieve a sense of justice by requiring individuals to bear such pain?” [21] Lavazza also argues that recognizing an individual’s unrestricted autonomy to erase memories may ultimately undermine both the autonomy of others within the community and, in turn, the individual’s own interests. If trauma victims were to selectively erase their painful memories, society as a whole might become indifferent and fail to uphold social justice [49].

However, this view may overlook two critical issues. The first concerns the definition of collective well-being. Attempting to weigh the benefits of improved mental health resulting from the erasure of traumatic memories against the harms of failing to fulfill duties of justice is inherently difficult to quantify. In addition, the concept of collective well-being could be potentially manipulated by certain ideologies, for example, by justifying hatred embedded in collective memory. The second issue relates to the neglect of individual well-being, which may even implicitly undermine fundamental human rights. For example, the European Convention on Human Rights states that citizens have the right to require the state to avoid actions that harm their mental health [50]. If an individual suffers from a severe psychological illness due to traumatic memories, and the institution refuses to erase the painful memories on the basis of the duty of justice, it may constitute a violation of fundamental human rights, which in turn fails to uphold justice.

In summary, previous deontological arguments concerning memory obligations, whether grounded in the duty of knowledge, assistance, or justice, each exhibit significant conceptual and normative limitations. Although these perspectives seek to justify the moral obligation to preserve traumatic memories, they fall short of convincingly establishing its universality.

## The rule consequentialist perspective on the obligation to preserve traumatic memories

Another approach supporting the moral obligation to preserve traumatic memories is based on rule consequentialism, which argues that individual memory holds significant social value, and our moral obligation is to maintain and enhance this value. Within this framework, preserving traumatic memories as a rule maximizes the overall public good of society [18]. As DePergola II notes, “society has some interest in preserving some memories for the greater good,” which forms the core reason for the moral obligation to preserve traumatic memories [33]. Individuals have a moral obligation to maintain the social benefits that come with preserving traumatic memories, whereas erasing them may undermine this benefit and violate the goal of maximizing social well-being, as advocated by rule consequentialism [51]. Thus, erasing traumatic memories is seen as an evasion of obligation and morally unacceptable.^3^

Opponents of traumatic memory erasure often interpret the public interests of traumatic memories from three aspects: the common good of preserving traumatic memories includes public safety, psychological resilience, and collective identity. I argue that these reasons fail to demonstrate that there exists a universal moral obligation, and that erasing traumatic memories necessarily violates such an obligation.

### Public safety

Some argue that one of the public values of traumatic memories lies in their role in maintaining public safety, and therefore we have an obligation to preserve traumatic memories [49]. Adamczyk and Zawadzki summarize the position in favor of protecting public safety and opposing MET as follows: if every victim were to erase their traumatic memories, public safety as a common good would face serious threats [7]. This is because, once victims lose relevant memories, they may be unable to testify in judicial proceedings, potentially allowing criminals at large. While these memories may often be very painful, the argument is that we should still make necessary sacrifices in order to promote the common good, and thus the moral obligation to preserve traumatic memories may override the individual’s interest in erasing those memories [18].

However, as pointed out in Sect. The deontological perspective on the obligation to preserve traumatic memories, using traumatic memories of identifying criminals as evidence poses reliability issues. Additionally, this social value and individual well-being can be balanced by extending evidence to other media or by eliminating emotional memories while preserving semantic memories. Moreover, allowing offenders themselves to erase traumatic memories does not necessarily jeopardize public safety and might actually help reshape their value systems and improve their moral decision-making [56]. Empirical evidence also suggests that refraining from intervening in offenders’ memories may, in some cases, pose an even greater threat to public safety. For example, prisoners suffering from PTSD exhibit high rates of psychiatric comorbidity, which are correlated with suicidal behavior, self-harm, and aggression [57]. Furthermore, offenders with untreated PTSD during incarceration are significantly more likely to reoffend compared to those without PTSD, thereby posing ongoing risks to community safety [58]. Therefore, it remains uncertain whether maintaining public safety in fact maximizes social utility, which renders this argument a tenuous basis for defending a moral obligation to preserve traumatic memories within a rule-consequentialist framework.

### Psychological resilience

Another rule-consequentialist perspective supporting the universal duty to preserve traumatic memories argues that if preserving these memories can enhance psychological resilience, it would promote overall social utility. Psychological resilience is the capacity of individuals and groups to adapt positively to significant adversity. For individuals, traumatic memories can be reinterpreted through self-narratives, transforming them into meaningful experiences that foster personal growth and psychological well-being, expressing the enduring power of resilience [22, 59]. For collectives, psychological resilience enables the society to withstand potential future changes, especially those arising from social disruptions, cultural transformations, or collective traumas [60]. Erasing individual memories may produce “composition effects” on the collective psychological state [49]. The stability and cohesion of a community in the face of traumatic events may be sustained by individuals’ capacity for reflection and growth, while individuals’ ability to withstand trauma may, in turn, be reinforced through the community’s systems of mutual aid and mental health resources. For example, research shows that young refugees in the Netherlands, when facing past traumatic experiences, were able to strengthen their sense of control and contribute to collective psychological resilience by engaging in self-empowerment strategies and integrating into new societies [61]. Survivors of prolonged civil war, despite exposure to complex trauma, are able to experience post-traumatic growth, exhibiting stronger social value orientations [62]. Memory erasure may disrupt this dynamic, as psychological resilience depends on retaining memories that enable individuals and communities to learn from past experiences, process emotions, and cultivate adaptive coping strategies.

However, the issue lies in whether preserving traumatic memories actually promotes psychological resilience and social benefits, or whether it exacerbates individual suffering and social mental health crises. The effects are unclear. The root of the issue lies in the fact that the very concept of psychological resilience may itself be subject to scrutiny. In Zawadzki and Adamczyk’s analysis, the integration of traumatic memories into a coherent self-narrative and the subsequent self-definition as a process of personal growth requires the mobilization of psychological resources [59]. However, merely surviving trauma, accompanied by suffering but with only a minimally coherent self-narrative, does not necessarily constitute morally significant growth. Likewise, even when traumatic memories are integrated into a positive narrative, the persistence of negative neural imprints that elicit physiological responses associated with PTSD makes it difficult to assess the extent or moral significance of resilience.

Moreover, it remains unclear whether the social benefits of individuals cultivating psychological resilience through preserving traumatic memories outweigh those of individuals attaining psychological relief through memory erasure. Currently, no public mental health research has provided a firm answer to this question. Mohatt et al. point out that for collective traumatic memories of historical events, no clear psychological causal pathway has been found that directly links memories with psychological harm or resilience [63]. If the refusal to treat individuals with PTSD is made solely for the potential social benefit, it not only may represent an unjust choice for the individual, but could also lead to further deterioration in mental health, without genuinely promoting public good or social value [64]. Especially in the ethical stance that forcibly requires the preservation of traumatic memories, there seems to be a hasty commitment to the “value of suffering.” As critics have pointed out, “[t]he notion that we need to have suffering martyrs among us is cruel and exploitative” [25]. Therefore, given that the consequences of traumatic memories for psychological resilience are still unclear, we cannot directly conclude that erasing traumatic memories harms social interests or constitutes an act of evading moral obligations.

### Collective memory

Another rule consequentialist perspective argues that preserving individual traumatic memories can promote the formation and development of collective memory, which not only helps shape collective identity and a sense of social belonging, but also motivates group members to contribute to common goals [50, 65, 66].^4^ For example, traumatic memories, by preserving the lived experiences of Holocaust survivors, shape the collective identity of the Jewish people and contribute to broader collective practices of remembrance, empathy, and moral reflection across generations. Their social value lies in connecting the past with the future, rebuilding families and communities, and transmitting core moral and educational values [69]. Furthermore, some traumatic memories acquire new meanings over time, providing a foundation for understanding between groups through ongoing negotiation and reflection [70]. We have an obligation to uphold the social value of collective memory to ensure that it continues to play a role in promoting common good. Erasing these traumatic memories undermines these social values.

However, if a moral obligation is to be ascribed to every member of a community to preserve collective traumatic memories, its legitimacy must rest on public reason. The public reason for the obligation to preserve collective traumatic memories refers to the requirement that the obligation should be accepted without relying on the specific conceptions of good held by particular individuals or groups. If such a reason cannot be provided, then the belief that erasing traumatic memories harms collective identity and public interest is deemed unjustified [71, 72].

Vallier (2014) proposes three criteria for qualifying as public reason: intelligibility, shareability, and accessibility. Intelligibility means that the reasons should be understandable in public discourse, but it does not require that everyone agrees with these reasons. However, this standard is too lenient to establish a universal obligation. Simply understanding the reasons for an obligation does not necessarily make it universally accepted. Shareability emphasizes the need for all citizens to accept the public interest reasons for preserving collective traumatic memories within their subjective motivation set, and these reasons must be widely recognized. However, in a contemporary society with diverse values, it is inevitable that there will be disagreements among members of the collective. In this context, it is unrealistic to expect everyone to agree on the public interest of preserving collective memory.

Accessibility, as the third criterion for public reason, argues that public reason should be based on widely accepted evaluative standards and should be open to rational deliberation by all citizens, allowing diverse reasons to enter the public discussion. From the perspective of accessibility, the obligation to preserve collective traumatic memories should rest on a reasonable balance of political values, grounded in justifications that are acceptable to all members of society. For example, in the tension between supporting individual psychological well-being and preserving collective memory, Lavazza points out that if every individual’s autonomy to erase memories at will were universally recognized, it could lead to a scenario in which “everyone in the society will see their civic freedom affected in the long run,” with the virtuous seeking refuge from reality and wrongdoers evading accountability [49]. From the perspective of accessible public reason, such justification appears to support the duty to preserve memories as a necessary safeguard for both social cohesion and collective responsibility.

However, in a pluralistic society, the standard of public reason set by accessibility remains difficult to achieve. According to the principle of accessibility, any justification for either promoting or prohibiting MET on grounds of moral obligation must rely on evaluative standards that all citizens can rationally assess. Yet, when it comes to assessing which reasons may legitimately enter the “pool of justification” for clinical practice, accessibility faces difficulties particularly within a socio-cultural context characterized by value pluralism. For any obligation to preserve memory to qualify as a matter of public reason, accessibility must be realized across multiple dimensions, including content, form, and degree. This implies that even in specific cases, each dimension requires its own set of accessible justifications. For instance, it is necessary to determine which types of memories ought to be preserved, whether semantic, emotional, or all forms; in what manner they should be maintained, whether through external technological means or solely through personal recollection; and to what extent interventions should sacrifice individual interests in pursuit of broader social value. However, reaching evaluative agreement across all such points of contention proves exceedingly difficult in a value-pluralistic context.

For example, according to Lavazza’s reasoning, which takes the form of a slippery-slope argument, the widespread adoption of MET would be ethically unacceptable because it would entail substantial disruption to the social fabric as a whole. However, in a pluralistic moral landscape, such a slippery-slope claim may not be convincing to those who place greater trust in regulatory mechanisms or in society’s adaptive capacity. Likewise, individuals who regard MET primarily as a therapeutic tool and are less concerned about its potential manipulative applications, or who believe that deleting emotional memories while retaining episodic ones does not pose a problem within the slippery-slope framework, may also find this line of reasoning inaccessible. A single, monolithic slippery‐slope prediction thus fails to accommodate the diverse perspectives of relevant stakeholders or to capture the ethical nuances arising from different cultural contexts. Consequently, it is difficult to assert that a broad and general obligation to preserve collective traumatic memories lacks a solid foundation in public reason. It may not be necessary to preserve individual memories in order to maintain collective traumatic memory, nor to rely on collective memory to achieve the maximization of social values.

## Obligations to oneself from the perspective of transformative experience

### Obligations to oneself

Arguments defending the moral obligation to preserve traumatic memories within the frameworks of deontological ethics and rule consequentialism encounter significant challenges. However, past discussions on the obligation of memory have largely overlooked a crucial element — whether the erasure of traumatic memories violates an individual’s *obligations to oneself*. In this section, I argue that we do have moral obligations to ourselves, and that memory erasure, as a transformative experience, may in some cases enable individuals to better fulfill these obligations. It should therefore not be regarded as a morally impermissible form of neurointerventions.

In the Kantian ethics, “obligations to oneself” constitute a fundamental aspect of moral obligation, emphasizing respect for one’s own rationality and personhood [73]. Such obligations encompass both self-care and self-respect: while self-care requires us to acknowledge and preserve our own wellbeing, self-respect demands that we refuse to be treated merely as instruments for others’ purposes [74].^5^ From this perspective, certain bodily modifications, such as cosmetic surgery, tattoos, and piercings, may be argued to violate obligations to oneself [76]. These practices involve physical harm and pain and may also signify a rejection of one’s bodily integrity, thereby conflicting with the Kantian notions of self-care and self-respect.

According to some opponents of MET, memory erasure may lead to ethically problematic consequences by evading one’s obligations to oneself. Even traumatic memories may, over time, enhance an individual’s adaptive capacity, including the ability to learn from experience, to grow, and to cultivate personal development. To erase such memories merely because of the emotional distress they cause in the present is a short-sighted act. When similar situations arise in the future, these unresolved experiences may trigger renewed emotional turmoil, ultimately impeding personal growth, happiness, and one’s relationships within the community [59]. Therefore, the act of erasing memories cannot be regarded as a legitimate form of self-care. By undermining the very capacity for self-reflection that grounds moral agency, it constitutes a violation of one’s obligations to oneself [77].

The position outlined above reflects a classical line of reasoning, asserting that MET compromises an individual’s capacity to meet obligations to one’s future self. However, the soundness of this claim remains open to question. One challenge may arise at the conceptual level: the notion of “obligations to oneself” has been argued to involve certain theoretical difficulties, since obligations are typically conceived as binding and non-waivable, whereas self-directed obligations appear, at least prima facie, to be voluntarily relinquishable. For instance, while I may have an obligation to care for myself, I can choose not to fulfill it [78]. Thus, it may be argued that such obligations to oneself are neither mandated nor existent.

However, I would argue that this view does not convincingly negate the existence of such obligations. In fact, obligations to oneself are not necessarily subject to arbitrary renunciation. Certain self-directed obligations, such as the obligation to cultivate one’s own talents, may be voluntarily abandoned; yet those obligations grounded in one’s own welfare and interests, such as the obligation not to commit suicide or to avoid self-degradation, are far less easily relinquished. If individuals were allowed to renounce their obligations merely according to their own will, such as the obligation not to harm themselves, this would imply a troubling symmetry whereby others, too, might be permitted to cause harm. Therefore, except when such decisions arise from careful deliberation and moral reasoning, for example when weighing the value of an individual life against collective interests, the arbitrary dissolution of self-directed obligations should not be permitted [79]. Releasing oneself from one’s own obligations, even if possible, is by no means easy. Conversely, the very idea of a “waivable” obligation raises the question of whether it remains a genuine obligation at all, or simply a metaphorical extension of other moral principles [80]. Consequently, it is difficult to determine whether relinquishing one’s obligation to preserve one’s own memories can be morally justified.

Building upon the recognition that there do exist certain obligations to oneself that cannot be waived at will, the subsequent question is whether MET necessarily poses an absolute threat to such obligations. This, however, still need not be the case. In fact, not only with respect to the future, but also within the temporal dimensions of the present and the past, MET may function as a means of fulfilling one’s obligations to oneself.

### Refuting the future obligation argument

The Future Obligation Argument posits that we have an obligation to our future selves to promote our well-being and prevent harm. Consequently, this suggests an obligation to preserve traumatic memories, as erasing them could potentially cause harm to our future selves. This argument supports the notion of second-personal reasons for obligations, suggesting that obligations to oneself arise from the present self’s commitment to the interests of the future self. Schofield illustrates this second-personal view with an example of a smoker’s obligation to refrain from smoking [81]:

Early on, the smoker occupies a perspective from which she enjoys the pleasures of tobacco. Call this perspective “P_1_.” Later, she occupies a perspective from which she suffers from emphysema. Call this perspective “P_2_.” From P_1_, the smoker could judge that she would, were she presently occupying P_2_, issue a justifiable demand that she quit smoking [81].

Based on such a second-personal perspective, one might argue that preserving traumatic memories constitutes an obligation to the future self. A person P_1_ at *t*_*1*_, who strongly desires to erase a traumatic memory, must bear an obligation to care for their future self P_2_ at *t*_*2*_ after memory erasure. P_1_ may recognize that P_2_ could experience a loss of meaning or undergo an identity crisis due to the absence of those memories. Thus, if P_1_ at *t*_*1*_ were to imagine themselves as P_2_ at *t*_*2*_, they might raise a reasonable concern that traumatic memories should not be erased at *t*_*1*_. Therefore, it seems that P_1_ has a self-obligation to refrain from erasing traumatic memories.

However, this argument rests on a crucial assumption: that P_1_ can predict P_2_’s emotions and needs, or at least anticipate the possibility of P_2_’s regret. However, drawing on L. A. Paul’s theory of transformative experience, undergoing memory erasure entails both epistemic and personal transformation, meaning that P_1_ may be unable to foresee what P_2_ will actually be like or what P_2_’s value preferences will be [82]. For instance, while P_1_ might assume that certain memories are essential to P_2_’s identity, P_2_ may, through new experiences and reconstructed memories, develop a different sense of identity and values — one that no longer aligns with P_1_’s perspective. Consequently, the imagined demand from P_2_ in P_1_’s prediction that memories should not be erased cannot serve as a decisive reason for P_1_ to preserve traumatic memories.

Moreover, this line of reasoning could also justify an obligation to erase traumatic memories. P_1_ might, out of concern for P_2_’s wellbeing, decide to undergo memory erasure. Suppose P_1_ recognizes that the persistence of certain memories will continue to cause suffering or impede P_2_’s flourishing. In that case, P_1_ might conclude that erasing those memories is the best choice for P_2_, thereby fulfilling an obligation to promote P_2_’s wellbeing. This suggests that different individuals may prioritize different aspects of self-obligation: some may emphasize the duty of their present selves to minimize future suffering, viewing memory erasure as a form of self-care and a way of fulfilling their obligation to themselves; others may prioritize the duty to prevent their future selves from experiencing regret, thereby opposing memory erasure, also as an expression of self-obligation. Given this divergence, we lack a clear understanding on how the present self should align with the future self-care and self-obligation [83]. This fundamental disagreement arises from the transformative nature of memory erasure, which makes it impossible for the present self to predict the future self’s inner experiences and value preferences. As a result, the Future Obligation Argument fails to justify an absolute obligation either to preserve or to erase traumatic memories.

### The implications of transformative experience for obligation to oneself

The process of traumatic memory erasure constitutes a transformative experience. As the phenomenological and preference outcomes of such intervention cannot be rationally or precisely anticipated, individuals facing such a decision must critically reflect on the relationship between their present identity and their potential future selves, thereby assuming moral obligations to themselves as both who they are and who they may yet become.

The transformative nature of traumatic memory intervention also implies that this obligation to oneself is inherently dynamic. Memory erasure can trigger profound epistemic and personal changes, potentially redefining the normative relationships individuals have with themselves. This implies that obligation to oneself is not fixed but is shaped by the complex interplay between an individual’s memories, value system, and temporal selfhood. This transformative experience may reshape these relationships, altering the specific form of obligation to oneself. Given this potential for transformation, individuals must reconsider how to fulfill their obligations to themselves in the future in ways that accommodate transformative experiences.

In fact, our obligations to ourselves may extend beyond the anticipation of our future selves. They also encompass obligations to our past and present selves, arising from an individual’s understanding of their position and role within the transformative processes of selfhood.

Regarding our obligations to the past self, some may argue that erasing traumatic memories disrupts psychological continuity and even breaks personal identity. The present self who is about to make a transformative decision may no longer share the experiences and preferences valued by the past self. In this sense, the “new self” could be seen as betraying the “old self,” thereby relinquishing certain obligations. However, this argument faces three main challenges. First, a break in psychological continuity does not necessarily destroy diachronic moral obligations. Even if the subject of obligation is not the exact same self, a sufficient causal continuity can still ground self-directed duties.^6^ Second, obligations to one’s past self can be understood through the idea of narrative identity. Even if specific traumatic memories are intervened, the individual may still integrate the old self’s commitments into the meaningful structure of the ongoing life story, and thus continue to bear those obligations. Finally, if the past self’s value system is itself morally problematic, for instance self-destructive or shaped by internalized oppression, severing ties with it may represent a form of moral self-liberation. In such cases, using MET to betray the “old self” might actually be a kind of self-growth. The proper way to fulfill one’s obligation to the past self is to transform it. Therefore, freeing oneself from traumatic memories of the past does not necessarily mean abandoning one’s obligations to the earlier self. It may, in fact, represent a deeper and more responsible way of honoring them.

With respect to one’s obligations to the present self, some may argue that there is an epistemic duty requiring the current agent to make every possible effort to grasp the potential transformative outcomes that might follow memory erasure. Even though, when facing the challenge of a transformative decision, rational calculation or decision-tree methods cannot determine whether one ought to proceed with the use of MET or resist such intervention. The individual may attempt to approximate understanding through imaginative simulation, statistical data, or the testimony of others. Yet these methods are unlikely to yield certainty about how one’s personality and value would change after memory intervention. By contrast, the obligation to the present self is constituted precisely through the agent’s role as the decision-maker standing at the threshold of possible transformation, and therefore entails a continuing duty to care for oneself under conditions of uncertainty. Although the outcomes cannot be fully predicted or rationalized, the present agent must nonetheless sustain a form of self-care within the limits of their epistemic situation. This obligation does not demand complete knowledge of the post-transformative self; rather, it calls for maintaining a reflective and responsive stance toward one’s own potential transformative change. For instance, a patient deciding whether to undergo MET should remain sensitive to the transformative nature of the experience, aware of the possibility of self-transformation and the fragility of personal continuity, and mindful of the value of informed consent and autonomy in present decision-making. When facing the transformative decision of memory intervention, one’s present obligation lies in approaching potential transformation with prudence, humility, and openness.

Therefore, there are no universal natural duties, such as an obligation to maintain psychological well-being or an obligation to preserve traumatic memories. Instead, individuals have role-conditioned and value-based obligations to themselves that are grounded on reflective evaluation undertaken as their roles and values transform. Although the enhancement of individual well-being through memory erasure remains uncertain, this very possibility allows MET to serve as an opportunity for fulfilling one’s obligations to oneself rather than as a means of abandoning one’s duties toward future well-being. What ultimately matters is that through this process of reflective evaluation, individuals determine what best aligns with their understanding of self-obligation, which is bound together by their past, present, and future roles.

Moreover, such self-directed obligations should not be understood as a form of self-centeredness that neglects one’s social duties. It does not mean prioritizing intrinsic personal values while disregarding the obligations embedded in social relationships. In many cases, social obligations and obligations to oneself can be mutually reinforcing rather than dichotomously opposed. From the perspective of one’s moral roles, fulfilling obligations to oneself, such as undergoing memory erasure to enhance psychological well-being, may in fact enable individuals to better perform their social roles, for instance, by becoming more supportive family members or by carrying out their professional duties more competently. Conversely, attentiveness to social obligations may also prompt individuals to recognize and fulfill obligations to themselves.

A potential counterargument may concern the *unpredictability* of transformative experiences. Transformative experiences are, by nature, epistemically and evaluatively unpredictable, which may prevent individuals from rationally assessing the post-erasure phenomena and preferences, thereby reinforcing one’s obligations to oneself. However, one view may hold that the phenomenological consequences of traumatic memory erasure can be analogized to the psychological state prior to the traumatic event, or simulated through recalling experiences of natural forgetting. In this sense, memory erasure may not constitute a genuinely transformative experience but rather a predictable alteration. Consequently, within a decision context of certainty, the use of MET cannot be justified merely by appealing to one’s obligations to the self. For instance, an individual who can foresee their role as a witness ought not to prematurely relinquish certain obligations associated with preserving traumatic memories.

However, rejecting the impact of transformative experiences on obligations to oneself merely on the basis of reasoning for retrospection may be unwarranted. It is true that we often rely on our past experiences of forgetting or of living unscathed by trauma to phenomenologically and evaluatively assess our decisions, thereby discovering or constructing our values. Yet even with such retrospective models, it remains impossible to reach a determinate judgment as to whether preserving or erasing a memory constitutes, in phenomenological and moral terms, a good or bad outcome. In particular, the use of MET may induce profound transformations of the self, altering neuronal connections, disrupting psychological continuity, and fracturing narrative integration, rather than merely “deleting files” or rebooting a memory copy. Consider the following case, which draws inspiration from Lavazza’s analysis of METs in the context of warfare [85]: Josh, a journalism graduate with idealism, aspires to become a war correspondent, believing it to be a self-aggrandizing profession. Once on the battlefield, however, he witnesses the horrors of war, which shatter his convictions and leave him suffering from PTSD. Back home, tormented by these memories, Josh deliberates whether to undergo MET. He is uncertain whether, as a journalist, he has a moral obligation to bear emotional witness to such suffering, or whether erasing the traumatic memories would amount to a betrayal of his former conceited self. Even when he imagines returning to his pre-war mindset, he cannot be sure that this is a psychological state MET would actually restore, nor that he could genuinely become again the person who once regarded the brutality of war with detachment. This indeterminacy regarding one’s own roles and identity deepens the moral obligations one bears toward oneself. It prevents an individual from anchoring their self-conception merely in a fixed role such as someone who seeks fame or abhors war, and thus renders uncertain which specific obligations ought to be assumed. Consequently, one cannot simply discard the obligations owed to one’s past, present, and future selves. This condition reveals that across different decision contexts, an individual’s states and preferences are often incommensurable. Within the unstable structure of selfhood that MET may engender, the individual cannot anticipate the trajectory of self-transformation or the corresponding evolution of obligations to themselves, which must therefore be undertaken with deliberation and prudence.

## Conclusion

In rethinking the problem of moral obligation in preserving traumatic memories, the Obligation Evasion Argument faces numerous challenges and fails to provide a moral justification for a universal duty to preserve such memories. Individuals bear no duties of knowledge, assistance, or justice in this regard, nor does any public reason mandate the obligation to preserve traumatic memories. Intervening in traumatic memory, as a transformative experience, exerts unpredictable influences on the temporal roles and intrinsic values that shape one’s relation to the past, present, and future selves. Such unpredictability compels individuals to acknowledge obligations to oneself, fostering self-respect and self-care, thereby supporting the ethical acceptability of memory erasure technology. Moreover, this indeterminacy highlights how the diversity of individual value orientations shapes distinct life roles, giving rise to a pluralistic understanding of self-directed obligations. This may offer insights for clinical practice, including that effective communication between physicians and trauma patients regarding their personal roles is essential to achieving informed consent. Patients should be encouraged to examine their evaluative perspectives on self-directed obligations and to externalize certain testimonial commitments through the process of memory disclosure, thereby alleviating the burdens of traumatic memories and more fully realizing their obligations to themselves.

## Data Availability

No datasets were generated or analysed during the current study.

## Footnotes

1 It is necessary to clarify that these memory erasure technologies do not operate like wiping a computer’s hard drive or entirely eliminating memories as portrayed in science fiction films such as *Eternal Sunshine of the Spotless Mind*. Given the inherent complexity of memory, the more plausible mechanisms of these technologies involve reducing the likelihood that associated emotional cues are triggered, or disrupting the neural circuits underlying traumatic memories, thereby diminishing their subsequent retrieval. Moreover, although the causes of PTSD are highly complex and involve not only traumatic memories but also a range of physiological, environmental, and social factors, interventions targeting memory still represent a potential avenue for its treatment.

2 Regarding the source of the duty of mutual aid, Rawls argues from the principles of justice, maintaining that we have a natural duty to assist those in need, provided that doing so does not impose excessive costs on ourselves [13]. Other philosophers have defended the duty of mutual aid from the perspective of care ethics. For instance, Hans Jonas contends that such a social duty “can be seen as a framework under which care for the future of mankind is the overruling duty of collective human action in the age of a technical civilization.” [36].

3 One point that may need clarification here is why a consequentialist moral theory can justify individual moral obligations. Act consequentialism often faces challenges when addressing issues of duty. For example, act consequentialism might argue that if breaking a promise leads to greater utility than keeping it in a specific situation, then keeping the promise lacks moral justification and cannot constitute a moral obligation, which may conflict with moral intuitions. On the other hand, rule consequentialism offers an explanation for moral obligations by emphasizing that morality must be viewed as a system of public principles. If not keeping promises is accepted as a moral rule, the foundation of trust and cooperation in social interactions would be weakened, and the overall utility of society would decrease, with negative consequences outweighing the individual-level utility calculations. Therefore, rule consequentialism can explain why we have an obligation to perform certain actions from a perspective of overall utility [52, 53]. However, some scholars oppose the idea of rules as the source of moral obligations, which, at a metaethical level, forms an argument against the moral obligation to preserve traumatic memories [54, 55].

4 Past research on collective memory has focused on cultural studies and history, viewing collective memory as a socially shared cultural symbol or as a dynamic process of cultural production and consumption [67]. Recently, research in cognitive science has further revealed the unique nature of collective memory, pointing out that collective memory not only exists within individual memories but also organizes these memories through a shared mental model that spans across time and space [68].

5 In the *Groundwork of the Metaphysics of Morals*, Kant argues that we have obligations to ourselves and to others, and these obligations can be further divided into perfect and imperfect duties. In this stance, taking one’s own life violates the obligation to oneself (G: 4:421–3). In the *Metaphysics of Morals*, Kant states that without obligations to oneself, it would be impossible to fulfill obligations to others, and “there would be no obligations at all” (MM: 6:417) [75].

6 Derek Parfit argues that the moral significance of personal identity does not depend on strict numerical identity but rather on a relation of psychological and causal continuity that is sufficiently connected [84]. Even if an individual’s memories are partially erased, as long as this causal continuity is preserved, for example if one preserves the awareness of having undergone memory erasure, there remains enough connectedness between the former and the present self for one’s past obligations to continue along a coherent moral trajectory.
